# Let's Talk: African Caribbean Women, Mothering Motherhood, and Well-Being

**DOI:** 10.3389/fsoc.2019.00088

**Published:** 2020-02-21

**Authors:** Geraldine Brown

**Affiliations:** Centre for Agroecology, Water and Resilience, Coventry University, Coventry, United Kingdom

**Keywords:** African Caribbean mothers, intersectionality, health, well-being, mothering, motherhood, race, urban gun crime

## Abstract

In the UK, African Caribbean women's experiences of mothering and motherhood are often studied in isolation from how “race” structures and shapes their lives and everyday health and well-being. This failure to connect women's wellbeing and racialized injustices also occurs in debates around “gangs” and “urban gun crime” (UGC). The women's experiences and the effects on their health and well-being are absented from the discussions. This paper, based on my doctoral research, uses an intersectional analysis to explore the views of mothers engaged in community activities to limit “urban gun crime.” Like me, they defined themselves as of Black Caribbean heritage and we created “spaces” where we could “reason,” share experiences of womanhood, motherhood and mothering in a racist society and centre what is habitually ignored. They highlight the importance of getting mothering “right” with the stress and anxiety about the dire consequences of getting it “wrong.” They show their love, commitment and aspirations for their child/ren and the emotional and intellectual labor needed in their daily strategies to keep them from harm caused by state institutions and life “on road.” The research confirms the impact of “race,” racialization and racism to our lives and experiences of health and well-being and raises critical questions for the dominant and normative views of mothering and/ or motherhood that underpin UK policy and practice.

## Introduction: Auto/Biographies, Stories, and Social Science

Storytelling is a universal human experience through which we learn, maintain culture and community, and bridge collective realities with individual experiences (Bell, 2010, p. 131).

For me, Bell's epigram (2010) says that telling stories can stimulate what Mills (1959) called our sociological imaginations. Stories can help us to situate biographies within particular moments in the histories of societies. This process can reveal previously hidden viewpoints, connections, and influences to allow for their transformation. In this paper, I share stories of individual, familial, and communal health and well-being from the hidden viewpoint of Black mothers in the UK at the turn of the twenty-first century. I draw these stories from data gathered as part of my doctoral research from a group of professional Black women. Like me, they were mothers with a Caribbean heritage involved in community activism. The stories show that irrespective of our differences our lives aligned around our concerns for our families. This is not unusual, however, our challenges as mothers focus on how we see and meet a racialised structural reality intent on damaging our loved ones and ourselves. For us, as Black mothers, issues of health and well-being are grounded in our worries about what we have to do so that our children can survive and thrive in a society fashioned by a political system in which particular power structures of formal and informal rules, prevails (Taylor, 2009, p. 4). Our understandings of racism intersect with our experiences of mothering and motherhood.

This understanding also influences my choices as a researcher. It shapes what I work on, how I work, with whom I work and how I am accountable to the people I “research.” For me, “who I am” is central to all aspects of the research process, including the adoption of an auto/biographical approach for this paper. Although the approach has been around for a while, and despite recent developments within the social sciences, it is still the neglected approach that Letherby described (Letherby et al., 2013). Part of its neglect is due to its challenge to the understanding of science, objectivity and subjectivity. For Letherby,

All social research involves individuals both researchers and respondents who have subjectivities, who make subjectivities. Theorized subjectivity acknowledges that research is a subjective, power laden, emotional, embodied experience but does not see this as a disadvantage, just as how it is. Starting with subjectivity though does not mean that we shrug our epistemological shoulders and give into the subjective, indulging in our subjectivities. Rather, it requires the constant, critical interrogation of our personhood both intellectual and personal within the knowledge production process (Letherby et al., 2013, p. 80).

In this paper, “doing auto/biography” allows me to respond to a need to:

“…[T]ell it as we know it, placing our story within its history at the heart of race, and sing our own voices and lives to document the day to day struggles of Afro-Caribbean women…” (Bryan et al., 1985, p. 2–3).

For me, the “it” is the historical captivity of psychology, psychiatry, social policy and sociology to the interests of people who do not look like me and the uses of those disciplines and their scientific knowledge to justify exploitation, oppression and alienation (Rose et al., 1984; Bryan et al., 1985; Bulhan, 1985). Bryan et al. made it clear that they also challenged how women are marginalized in the African Caribbean community and echoing them Hill Collins (1990) challenges mainstream white feminism when she says that it is a feminist act to be accountable for the knowledge we produce and the actions we take. I am accountable to them.

This commitment requires an explicit recognition of the relationship between the process and the product, the knowing and doing relationship, as Letherby terms it (Letherby, 2003; Letherby et al., 2013). Harris (2003) argues that an auto/biographical approach can be a valuable way of revisiting aspects of our lives from a distance. It allow me to revisit our lives as Black women as a stranger to highlight the multiplicity of our identities and offers a vehicle to make my work more accessible (Mykhalovskiy, 1996; Letherby, 2003; Colombo et al., 2007). This illuminates the interplay between my position as a researcher, black woman, mother and the biographer of the women in my study.

The paper continues with the rationale and context for the study and then details issues around methodology. The findings are organized under three key themes and the discussion highlights how Black mothers' experiences challenge and enrich dominant conceptions of the “good” mother in early twenty-first century Britain. It concludes that these Black mothers' stories of mothering and motherhood cannot be understood without locating them within the wider system of racialised capitalism that structures and shapes their everyday realities of survival, health and well-being.

## Research Rationale and Context

My doctoral study explored the significance and meaning of “urban gun crime” for African Caribbean men and women engaged in community-based activity in two cities in the West Midlands, England. I did this for personal and political reasons. When I started thinking about the focus for my research in 2004, there was still media and political interest in Beverly Thomas and Marcia Shakespeare. They are the mothers of Letisha Shakespeare and Charlene Ellis, two black young women murdered in a drive-by shooting in Birmingham^1^. The killings occurred near my children's grandmother's home and not far from mine. As a Black mother, I felt those women's losses and feared for my own family members. As a researcher, I wanted to do something about the situation, to make it better through my work, my research.

The killings caused much anxiety locally and in other inner-city communities and provoked much comment across wider British society. Whilst the local concerns of the mothers and Black communities more broadly received some coverage, wider and whiter commentary shaped how such killings and “urban gun crime” itself would be accounted for and acted upon. Following the murders, according to the National Criminal Intelligence Service (NCIS), “urban gun crime” was repackaged from a key criminal justice problem to “a national crisis” [(National Criminal Intelligence Service (NCIS), 2003), p. 67] and mothers and communities' concerns about race, class and policing were lost as the Black family was pathologized for rearing cold-blooded killers even as they mourned their dead children.

In the UK, the term “UGC” is commonly accepted (often uncritically) and normalized within everyday discourse irrespective of the lack of overarching explanation and over reliance on specific forms of evidence from elsewhere (Hallsworth and Silverstone, 2009). At the time of the study, around three in five (59%) of such offenses were recorded in just three police force areas—Metropolitan, West Midlands and Greater London, with Black homicide victims concentrated in the same three areas. This reflects the geographical spread of Black and other Minority Ethnic groups (62% of Black people in England and Wales reside in these three areas) and the increased risk of being killed in urban areas (Hales, 2006). Consequently, my intention was not to deny the reality that there are young people from African Caribbean communities who are both victims and perpetrators, but to examine how “race” racism and a process of racialization are made visible or rendered invisible in experiences and discourses.

This shift in interest was a driver for my original research. I gave myself permission to reverse the usual gaze on Black families, that is, to turn the dominant narrative the right way up, to outline how Black families and mothers can offer new perspectives on and solutions to social problems. I aimed to redress the balance a little, to present the realities of Black families made responsible for the failings of Blair's Britain, to show how it feels and what it means to carry the blame for the “national crisis” of “urban gun crime” [National Criminal Intelligence Service (NCIS), 2003]. One objective was to analyse the crisis from both historical and existential perspectives as a Black mother and social researcher. Another was to explore the connections between the researcher and the researched as Black people, as Black women, and what we could learn as suggested by Bryan et al. (1985) when our experiences and material realities occupy center stage in the production of knowledge. Here, the everyday experiences of Black mothers and their children would be the foundation for the analysis of the actions of local and national states and the promotion of greater reflexivity and responsibility amongst social researchers.

## Theory: Intersectionality and the Heart of the Race

Building on the conclusions of African Caribbean women and engaged Black socialist social scientists in the UK, Africa, and the Americas (Davis, 1981; Bryan et al., 1985; Bulhan, 1985), Hill Collins (1990) asserted that the politics of “race,” class and gender influence the private and public production of knowledge. However, she argued that this is often done “unconsciously” by those within racist, capitalist and patriarchal structures whose agendas serve to manage and control Black communities. For her, most research and knowledge are manufactured to sustain existing structures, processes and positions and, given that, progressive politics need to re-place the intersection of racism, sexism and class exploitation at the center its analyses, especially when researching Black communities.

I used Hill Collins' position to modify the Black British working-class feminism I had developed based on my experiences growing up in Birmingham during the 1970s and 1980s, my working life as a teacher and social researcher and the scholarship of writers such as Sivanandan (1985); hooks (1989); Crenshaw et al. (1990), and Bryan et al. (1985). This allowed me to think reflexively and publicly about both my lived experiences and those of others like me in the UK and the wider systems of discrimination that impact Black people's lives (income, education, housing, employment and crime) (see Khan and Shaheer, 2017) and wider sets of ideas ignored by mainstream discourses (Hill Collins, 1990, p. 7). These were the foundations for the counter narrative to the prevailing racialised and gendered discourse around “urban gun crime”.

## Methods

For my methods I drew on Harvey (1990) critical ethnography approach, which advocates the use of “tried and tested” ethnographic tools for new reflexive practices and ends. These include attending to how participants' lives are situated within and constrained by structural socio-historical factors, such as African Caribbean women's understandings and experiences of a “maddening” white society (Bulhan, 1985). Of course, there were issues around reflexivity and my positioning within the process to be considered, especially as my field work was carried out in two areas of the West Midlands that I knew and where I and had access to relevant networks. My previous work in policy-oriented qualitative research had both attuned me to these concerns and provided me with a simple solution based on collaboration, transparency, communication, and relationships (Ali et al., 2007; Brady et al., 2012).

Given that, my principal method was overt participant observation. As a researcher, I would be an active and reflexive participant in the lives of the people I researched. This allowed some satisfaction for my political, personal, and scholarly concerns. There is much debate in the critical ethnographic literature about such engaged research, the details of which need not detain us here. Suffice to say that the issues were discussed in depth and detail with supervisors, advisory teams, contacts with the community groups and ethics committees. This approach allowed me to contact a community-based initiative in each area, to declare my interests both academic and personal and to offer my skills, knowledge and expertise to support their work as a volunteer. Possible ethical issues and potential conflicts of interest were noted at these meetings and as I became aware of them they were discussed and dealt with at the earliest opportunity with relevant stakeholders.

In the doing of the research I was aware that the ethical challenges researchers encounter goes far beyond what we are required to consider when we complete our ethical approval application and get institutional consent to proceed with our studies. There are a plethora of issues that can arise in the field, during our analysis and in the reporting of our work. It is impossible to predict all of them. Research is not a linear process, but messy and dynamic; irrespective of the time spent planning and organizing, research can be unpredictable and ad *hoc* (Hammersley and Triaianou, 2012; Brown, 2015). Given this, researchers need to make reflexive judgments about the research situation, including the identity of the researcher and of the “researched” and the forces of various kinds, operating upon and within it.

## Participants

The areas selected for my study had higher than average African Caribbean populations and had been the targets of police-led concerns and community-led interventions around gun-related incidents. I volunteered and collected data over a period for 22 months. This paper draws on conversations and interviews with five mothers, who have been assigned pseudonyms (Susan, Alison, Rachel, Angela, and Shirley), professional women aged between 35 and 60 years old involved in local community work, whom I met at community events. Only Susan was recruited due to her involvement in the community group I observed for the study.

## Women Included in the Study

**Table:** 

**Pseudonym**	**Age**	**Profession**	
Susan	45–50	Civil Servant	Susan lived in Area 1. She was born and grew up in the West Midlands. She spent time as a single mother and at the time of the study worked as a civil servant and local magistrate. She was an active member of a community-based organizations with a specific focus on “urban gun crime.”
Alison	50–55	Minister	Alison lived in Area 2 and was married with children. She had been a local vicar for over 25 Years and was based at an inner-city church. She was not directly affiliated to a community-based organization, but was an activist engaged in a range of community activities.
Shirley	35–40	Probation Officer	Shirley was born and lived in Area 1. She was employed as a Probation officer, and was married with a child in secondary school. Shirley was not affiliated to a community-based organization but was a practicing Christian and active in her local community.
Angela	55–60	Health Practitioner	Angela lived in area 1. She came to the UK at 14 and spent most of her time here living and working in the West Midlands. She trained as a nurse in the 1970s and is a mother and grandmother. She managed a community project to meet the mental health needs of African Caribbean communities.
Rachel	45–50	Youth Worker	Rachel lived in Area 2 and was born in the West Midlands. Rachel has four children and raised them on her own from teenagers. She was employed as a local authority youth worker and had set up a community group for parents whose children have been involved in UGC and/ or “gangs.”

## Data—Analysis

Ethnographic research sets great store by the gathering of stories through interviews and informal conversations. Issues around the trustworthiness and credibility of respondents and the researcher are much debated (Harvey, 1990; Coffey, 1999). Interviews were recorded and transcribed verbatim with the aim of generating theory using a grounded theory approach (Glaser et al., 1967). This would be faithful to the data and acknowledge that researchers do not enter the field with “empty heads” (Stanley and Wise, 1993). I used a system of “open coding” that sorted the data into analytical categories by “breaking down, examining, comparing, conceptualizing, and categorizing” it (Strauss and Corbin, 1990, p. 61). These categories were then compared to generate the themes. Three themes are presented in this paper; namely: the importance of doing mothering and motherhood “right;” the daily stress and anxiety and costs of getting things “wrong” and their strategies for safeguarding their child/ren from coming into harmful contact with both state institutions and “life on road” (Glynn, 2014).

## The Importance of Doing Mothering and Motherhood “Right”

The themes and the stories that inform them show the contradictions of the normative view of mothering and/ or motherhood dominating UK policy and practice and the significance of racism to Black mothers' lives and experiences of health and well-being. The mothers' views and experiences redefined the common role of a “good” mother to include providing a nurturing and caring home for their child/ren and preventing them from becoming “known” to the state and its institutions.

Rachel, the youth worker, had a son in prison for a gun related crime. She explained that, her choice of career was partly in response to her son's first involvement with the criminal justice system following a fight with a white youth whose father was a neo-Nazi. She said:

At the time was I was going through a very bad relationship with my children's dad and my son started to get involved with the police. He had a fight with a white lad whose father was in the National Front. They used to come down on bikes and torment the kids. My son started to fight and before you know it, I had to take my son to the police station. At the time he was about 12 or 13 years old and they read him his rights. They tried to charge him with Grievous Bodily Harm (GBH). In those days I wasn't that educated about the system as I am now. From then on, my son started to experience problems in his life.

Rachel felt she had failed her son, that he was now merely a statistic, another young black male who had not completed compulsory education, because he was excluded from school, identified as a “gang” member, convicted and serving a lengthy prison sentence. His negative relationship with the state and its institutions were a barometer for her sense of failure as a mother. She feels herself to blame for failing to keep him safe from the system, even for his reading difficulty:

So yes, I have to take some of the blame. Maybe, I should have helped with his education. He wanted to work. He wanted to learn, but he had to start from the beginning. He had to read those little books and the system failed him…

All the women spoke about the importance of their role as mothers and the pressure they experienced doing what they believed was in their child/ren's best interests. They saw paid work as both a positive example for their child/ren and a necessity to meet their needs. Overturning the stereotype of the welfare dependent single Black mother of criminal children, Rachel said:

I did not want to be just a mum who went to the post office got her money and came home and sat down. I call it “The Government Routine”—it was like they mapped out your life. So, I studied and did a lot of community work.

Alison, a married vicar, lived in a part of the city with a small African Caribbean community and she spoke about the unintended consequences of sending her son to a “good school” in a “good” neighborhood. Here “good” is racialised and carries its own problems, as she says:

It is very easy to beat oneself up X number of years down the road, but when I look at my son now and see the damage that it did to him by taking him to a White school. It was misguided on my part anyway and it will probably take a long time for me to forgive myself for that, even when you think you are doing right thing!

Alison was also clear that she felt that Black mothers had their authority undermined by the state on matters of familial discipline. Again, the stereotype of the overly authoritarian Black mother is questioned by Alison's more nuanced questioning and observations about consequences.

Parents have been disempowered because of legislation and no one is talking about brutalizing children because it is not the thing to do, but parents are worried, and children know their rights and threaten parents with bringing the authority. Should they speak too harshly? Should they raise their hands? I think we reap the benefits of that particular movement. If children have rights, parents have rights too. And, if parents are devoid of the opportunity to appropriately discipline their children, there are going to be problems and the statutory agencies take them under their wings. So, you see what you see.

Where Alison was worried about the impact of her wrong decisions and powerlessness on her son, Angela, with adult children and a grandchild, spoke of her fears for the future, even if she did everything right. Angela said:

I worry for my children who are in their late twenties. I worry because I don't know what sort of future there is for them, especially my granddaughter. You try to protect them from all sorts and you can do the right thing and they can still get caught up in stuff and that is why I cannot buy into its necessarily solely about single Black women bringing up their children on their own. Boys becoming men. Oh God! This is depressing!

As Angela shows, the mothers felt overwhelmed by their fears about gun crime. They felt a weight of responsibility, anxiety and powerlessness. They felt it was incumbent on them to do all they could to prevent their child/ren from getting involved in “life on road” and/or with the state and its institutions. “Urban gun crime,” regardless of their familial proximity to it, was a defining reality for these African Caribbean mothers. Shirley's work as a probation officer brought her into daily contact with young Black men “criminalized” by the system. She spoke of how her work shaped her discussions with her son and how she managed her daughter's school exclusion:

I always say to my son that, he is 13-14 going thorough whatever happening or schoolteachers, you know, don't like him. It's the same thing, you're not going to like everybody and not everybody is going to like you. So, I say, the thing is that you're a boy but you're Black first and the expectation is that you're going to fail. So, trying to work with that psychology and showing him the opposite and that actually he can achieve but not everybody has that same kind of input. I believe, again talking about an experience with my daughter, she was excluded. If it was left to the authorities, she would have been sent to place for children with behavioral issues. But I said no and went and did my own research and found an alternative place for her to get her education and the actual social inclusion officer she didn't like that because I went and did my own research, but not every parent would do that.

The mothers' recognition of and responses to racist institutions shaped their experiences of mothering and their understanding of a “good mother.” Their efforts to be “good mothers” redefined how they understood and experienced their own physical and mental well-being—it was enough for them to survive if their children could be kept safe from the state and the road.

## The Daily Stress and Anxiety and Costs of Getting Things “Wrong”

When Rachel could not keep her son safe from the law, she felt it was even more of an imperative to support him, to do her best to stand by him. She illustrates the stresses of good Black motherhood. She said:

I've sat in courts. I have been harassed by the police. They have even pointed a gun at my head. They started to come to my house with guns.

I reached a point where I nearly had a nervous breakdown. I would be sitting up all night. …They came one night, “Can we come in?” As soon as [her son] came downstairs, they arrested him.

The mothers were clear that they feared such outcomes and that the fear took its toll on them. Even dealing with the fear affected their mental health. One said she prepared herself for being told that her son was dead, hospitalized or imprisoned. Other mothers dealt with this fatalism by refusing to attend courts or visit their sons in prison. They cared, but their refusal to be present was a way to deal their powerlessness by turning away from it. They knew that they were not “good mothers” but felt that they had to get on with the rest of their lives. They could not win and had no choices.

However, Rachel spoke about her efforts to give her children choices even in difficult times.

He came from a very structured home, but because of his lack of education he had trouble in school. This is a problem I see with our young Black men. Schools if you don't achieve, they try to get rid of you because of their stats. My son had a problem that he couldn't read, and he got teased by the other kids. In the end, they threw him out of school. They said they would pay for him to go to college instead, but they just sent him home. The streets were just looking for him.

Like Alison, Rachel identifies the key role of education in shaping Black children's lives and the health and well-being of their mothers and carers. Rachel's son was simply sent home from a “normal” school because of his reading difficulty and the street was there looking for him, whilst Alison risked her son in a “good” school in a “good” area for fear of that same street. She said:

I did not want to live in the inner city partly because I had my children and I was afraid about what I heard about as gun crime and did not want my two boys to be dragged in this, because they are very gentle personalities and I think they would have been quite overwhelmed, not because I didn't want them to be amongst my own people, but I didn't want them to get caught up in gangs.

Where Alison had choices about where she lived, Susan said that some mothers have no choice but to work and be away from home and their children. It can cause problems when “Parents are out at work busy trying to make ends meet, it is a vicious circle.” Another aspect of the “vicious circle” can be the mothers' intimate relationships, the presence of men in their families' lives and contradictions of male children becoming “the man of the house”. Angela notes:

I think that quite often black women are trying to be mother and father and sending mixed messages and sometimes the lines are very blurred because she start off by saying that he is the man of the house and he's not he's a boy and when she tries to flex her muscles she has already planted the seed in his head that he is a man. Not all black women do that there are some Black women who successfully raise boys… but I think there is also this thing about the support form male family members.

She highlights the difficulties of renegotiating familial roles in times of stress. Such factors are overlooked when discussions link “urban gun crime” to the family by way of a focus on absent fathers (and, by extension, failed mothers). The women were clear about the differences between the realities of their lives and this dominant discourse. The presence of fathers and male partners can also be a source of stress and ill-health in a racist society. As Alison says:

I just get really angry when Black men begin to beat Black women up, as if we're not beaten up enough by society and, when they start to do it, we're in trouble. So, I think, it seems to me, if we are going to move away from this impasse, this sort of …

We're in the trenches aren't we and the other one is over that side and we're over here and we're cursing each other like hell. It seems to me, if we are going to get beyond that, I think we are going to have to sit down and talk, because I don't think it is helpful for Black men or Black women to beat each other up. That is exactly what White people want us to do!

The mothers did not romanticize Black motherhood but knew that poor parenting was not the monopoly of Black women and their families. For Alison:

I made my mind up and I started thinking about this long before I came here that there are some people who should not be allowed to have children, partly because our society has become amoral or immoral and that just affects everybody. And I see kids behaving badly Black and White. Why? …It could be that the father is not at home, but that is not only reason because my father died, but my mother kept it together. I think it is too easy and too simple to make Black women feel bad that their kid has gone off the rail.

Alison's observations contain challenging ideas. She sees a general decline in the quality of behavior and Black young people's involvement in crime as part of that. Her radical solution was born of despair.

## “Race” Matters

Underpinning the women's conceptions of “good mothers” is the ability to nurture and protect in a racist environment in the UK. The costs of failure (or, even, success) can be swift and unforgiving—a child is shot or imprisoned—or chronic and debilitating—dealing with the long-term consequences of stress and provocations to breakdown. The women were aware of relationships between their everyday experiences and the intentions and actions of the state and other bureaucracies. They were also clear about changing the nature of this racist environment.

Angela said that in the 1960s and 70s, she experienced “open racism.” It was explicit and unapologetic and a part of everyday life. It encouraged community responses and political and social activism felt easier. Contemporary racism was for most far subtler, for her, but no less harmful:

Now that certain words aren't politically correct, it doesn't mean that the impact is any different or that it has gone away, but it is just that it is more covert now, you know. You are feeling the effect!

You go somewhere and you pick up the vibes and you look at what happens with our children in schools. It is still there, it is not as blatant, but the impact it is worse and, because people can't see it, and a lot of our young people, when you talk to them about racism, they don't think it exists because people aren't in their faces calling them the “N” word or you know this kind of thing.

So, they don't understand. I think that makes it a bit more difficult.

Angela saw what she called institutional racism as more difficult to identify, organize around and challenge. She thought that this would have major ramifications for young people and their ability to challenge the system that shapes their lives:

A lot of our young people, they think, I go to school and sit next to other people and they are alright, but they don't understand the structural nature of racism and how it dictates where they go, how they can go, what they do. The fact that they are disproportionately included in school exclusions and within mental health system, prison system because 40% of the prison population are young Black men. They don't understand that!

Although a lot of our young men are bright, sadly they don't have the analytical depth to even be able to see anything beyond face value, because schools don't teach them to think, but they can regurgitate certain things.

Susan also notes the “disappearance” of institutional racism from statutory bodies' agendas and their inability and unwillingness to consider effective strategies to promote real change:

I think the statutory bodies want to, because it is easier, take a simplistic approach in that they want to deal with the symptoms as oppose to the causes. Because the causes are long standing, well entrenched in the “very difficult to do” box, so treating the symptoms is going to appease the statutory bodies in that they are doing something. What that something is and how that impacts and the longer standing issues that is the question.

For these women, the actions of the state and its institutions have long-lasting effects on limiting the well-being and freedom of their families.

## Final Reflections: Absent Mothers: Let's Talk About their Health and Well-Being

The application of an intersectional analysis is a means of capturing and making visible the relationships between multiple systems of power in the lives of Black women (Hill Collins, 1990; Crenshaw, 1991, 1989). It shows how our experience of mothering and motherhood is mediated by a matrix of power (economic, political, cultural, and experiential). As I reflected on the research themes, I realized that most discussions around “gangs” and/ or “urban gun-crime” and its consequences focuses on the presence and/or absence of men. Black women's experiences only appear in relation to those of men and their well-being—Black mothers' lives do not matter, except in relation to what their sons and baby-fathers do. The roles and experiences of Black mothers and the challenges they face in their mothering, such as navigating intimate and familial relationships, violence, work and economic challenges as they are compounded by institutional racism are absented.

To date, the impact on Black mothers' health and well-being has not been appropriately considered. In 2013, Bailey et al. highlighted institutional inattention to the emotional consequences for losing or having a son injured due to “urban gun-crime” or police operations. The focus is usually less on the “actual” impact on the families of perpetrators, victims and bystanders than the “perceived” assault on British values by the murderous character of inner-city Britain. As my respondents suggested, mothers are often traumatized by both the actual events and the fear and trepidation surrounding them. As they have experienced armed police in their homes or mourned in advance, they find it difficult to find meaning in what has happened to their children and the society that shaped them. Lawson (2014) says that their actual grief and their preparations for it are also invalidated by that society and undermined and dirtied by police practices.

In telling their stories, these mothers show that “the political is personal” (Mills, 1959; Bourne, 1983). For them, debates about “urban gun-crime” are not out there but play out in their everyday lives in multiple ways. Their observations question the so-called “objectivity” of commentators, politicians and policymakers who mask the realities of their lives and the histories of their communities. These mothers felt that discussions around “gangs” and “urban gun-crime” were how the powerful, the polite and the racist aired their real views about Black communities. The terms were simply euphemisms for old discussions around the pathology of the Black family. These mothers illuminate how their experiences are often understood in isolation from the role of patriarchy, race, class, ethnicity, and age and the other variables which structure and shape our experiences of the social world. Nonetheless, the idea of the chaotic and dysfunctional Black family structure and ill equipped mothers is powerful and it is this image that is often presented within the public domain and repeatedly used as an explanation for a range of social problems encountered by African Caribbean communities sustaining a lack of focus on issues associated with health and well-being.

## Data Availability Statement

The datasets for this study will not be made publicly available because at the time of the study participants were informed that only the researcher and supervisory team would have access to interview and observational data and this formed the basis of their consent.

## Ethics Statement

This study was approved by Coventry University Ethics Committee and carried out in accordance with the guidelines set out (ethics.uni@coventry.ac.uk). All participants gave written informed consent. The data is anonymized and participants assigned pseudonyms.

## Author's Note

This paper is based on my doctoral research. My research was conducted in two localities in the UK and explored how men and women who identified as African Caribbean and engaged in community activism conceptualized and responded to “urban gun crime”. My thesis, presents an analysis of participants' conceptualization of “urban gun crime”, community activism and consideration of my subjective insider/outsider positionality in the production of knowledge.

## Author Contributions

The author confirms being the sole contributor of this work and has approved it for publication.

### Conflict of Interest

The author declares that the research was conducted in the absence of any commercial or financial relationships that could be construed as a potential conflict of interest.

## Footnotes

^1^Birmingham is the second-largest city in the United Kingdom with roughly 1.1 million inhabitants within the city area and 3.8 million inhabitants within the wider metropolitan area.
